# The role of intestinal microbiota and its metabolite short-chain fatty acids in hypertriglyceridemia-associated acute pancreatitis

**DOI:** 10.3389/fmicb.2025.1667075

**Published:** 2025-11-05

**Authors:** Qing-Qing Guo, Xiao-Dan Wu, Hao Lin

**Affiliations:** ^1^Department of Intensive Care Unit, the First Affiliated Hospital, Fujian Medical University, Fuzhou, China; ^2^Shengli Clinical Medical College, Fujian Medical University, Fuzhou, China; ^3^Department of Anesthesiology, Fuzhou University Affiliated Provincial Hospital, Fuzhou, China; ^4^Department of Gastroenterology, Fuzhou University Affiliated Provincial Hospital, Fuzhou, China

**Keywords:** intestinal microbiota, gut homeostasis, hypertriglyceridemia-associated acute pancreatitis, inflammatory response, short-chain fatty acids

## Abstract

Hypertriglyceridemia-associated acute pancreatitis (HLAP) is a severe gastrointestinal condition characterized by an increased risk of multiple organ dysfunction and elevated mortality. Intestinal microbiota, often described as the second human genome, plays a key role in maintaining gastrointestinal and systemic homeostasis. Among its various metabolites, short-chain fatty acids (SCFAs) are particularly abundant and functionally significant. Current evidence indicates a strong relationship between SCFAs and the pathogenesis and progression of HLAP. SCFAs contribute to the restoration of intestinal homeostasis by modulating the composition of gut microbiota, enhancing the integrity of the intestinal epithelial barrier, and regulating mucosal immune responses. Furthermore, SCFAs attenuate systemic inflammatory responses, promote pancreatic tissue repair, and reduce the risk of multiple organ dysfunction. These protective effects indicate that SCFAs represent a promising therapeutic target for gut-centered interventions in HLAP. This review summarizes the changes in intestinal microbiota and SCFA levels following HLAP onset, elucidates the underlying mechanisms by which SCFAs exert protective effects, and evaluates their potential therapeutic applications, thereby providing a theoretical basis for the development of gut-targeted strategies in the management of HLAP.

## 1 Introduction

Acute pancreatitis (AP) is characterized by acute inflammation and cellular injury within the pancreas and is recognized as a common cause of acute abdominal disorders. With improvements in living standards and shifts in dietary habits, the incidence of hypertriglyceridemia-associated acute pancreatitis (HLAP) has significantly increased, surpassing alcoholic pancreatitis to become the second leading cause of AP (Chinese Pancreatic Surgery Association, and Chinese Society of Surgery, Chinese Medical Association, 2021). Additionally, HLAP is increasingly observed in younger adults and is associated with severe clinical presentations, including a higher incidence of complications such as acute respiratory distress syndrome (ARDS), acute kidney injury (AKI), and multiple organ dysfunction syndrome (MODS) (Li et al., 2018). Increasing attention has been directed toward the role of intestinal dysfunction in the progression and exacerbation of HLAP, particularly in relation to gut microbiota imbalances, compromised intestinal barrier integrity, bacterial and endotoxin translocation, and systemic inflammatory response syndrome (SIRS).

Patients with HLAP commonly present with changes in gut microbiota diversity and composition, notably an overgrowth of pathogenic bacteria and a reduction in beneficial microbes, especially those involved in the production of short-chain fatty acids (SCFAs). SCFAs including acetate, propionate, and butyrate are predominantly produced through microbial fermentation of undigested carbohydrates and glycoproteins secreted by intestinal epithelial cells. These metabolites function as secondary messengers that facilitate signal transduction and influence disease progression, primarily via two mechanisms: inhibition of histone deacetylases, which elicit epigenetic effects, and activation of G protein-coupled receptors (GPCRs), which mediate downstream signaling pathways (He et al., 2020).

As metabolic byproducts of the gut microbiota, SCFAs serve as an essential energy source for intestinal epithelial cells (Yang et al., 2023; Pouteau et al., 2003). A portion of SCFAs is absorbed into the circulation and transported to hepatocytes and adipocytes, where they contribute to glucose and lipid metabolic processes (Niccolai et al., 2019). Beyond their metabolic roles, SCFAs are key regulators of intestinal barrier integrity and immune function. They contribute to the preservation of the mucosal barrier by modulating the expression and localization of tight junction proteins and enhancing mucin secretion on the intestinal surface (Wang et al., 2012; Willemsen et al., 2003). Additionally, SCFAs suppress the production of pro-inflammatory cytokines and facilitate the recruitment of immune cells, thereby modulating both local and systemic immune responses.

**Figure 1:**
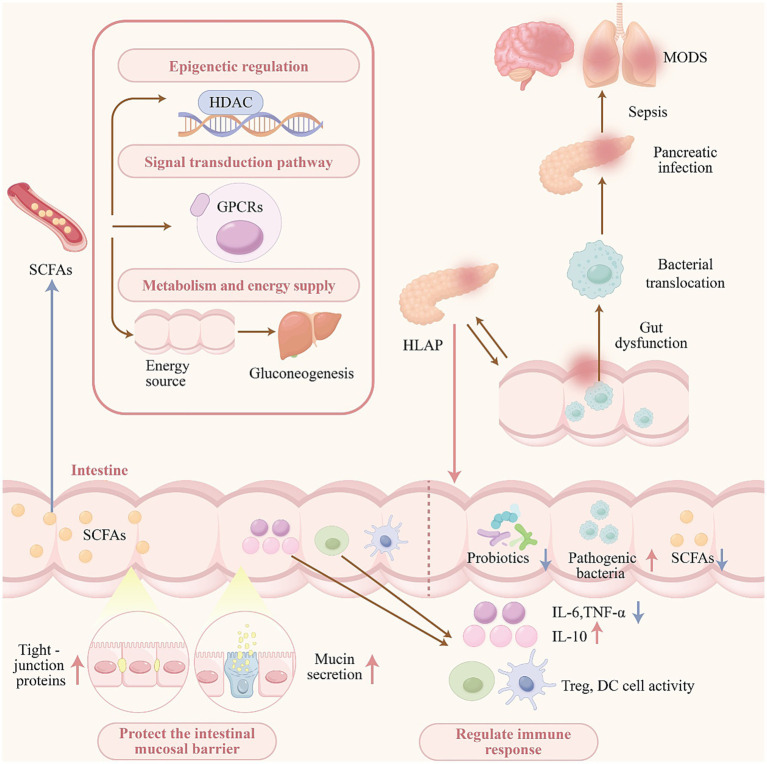
Gut dysbiosis and reduced SCFA levels in HLAP promote disease progression.

In summary, the observed dysbiosis and reduction in SCFA levels among patients with HLAP are associated with compromised intestinal barrier function, increased bacterial translocation, pancreatic tissue infection and necrosis, and an elevated risk of sepsis and MODS (Li et al., 2020). These findings highlight the potential of SCFAs as key therapeutic targets for future HLAP interventions (see Figure 1).

## 2 SCFAs—an overview

SCFAs are saturated fatty acids containing one to six carbon atoms and are primarily produced through the anaerobic fermentation of undigested and unabsorbed carbohydrates, predominantly resistant starch and dietary fiber as well as glycoproteins secreted by intestinal epithelial cells in the colon (Facchin et al., 2024). Among the SCFAs present in the gut, approximately 90% consist of acetate, propionate, and butyrate, typically occurring in a molar ratio of 6:2:2 (Rooks and Garrett, 2016). The sources, distribution, and physiological effects of SCFAs on the host vary depending on factors such as the composition and abundance of gut microbiota, the origin of fermentable substrates, and intestinal transit time.

Acetate, the most abundant SCFA in the colon, is produced by a wide range of *Enterococcus* spp. It is readily absorbed and transported to the liver, where it primarily contributes to lipid and cholesterol synthesis, and serves as an energy source for peripheral tissues (Li et al., 2018; Zhang et al., 2023). Propionate, generated by bacterial species including *Bacteroides*, *Acidaminococcus*, and *Salmonella*, is mainly absorbed by the liver and utilized as a substrate for gluconeogenesis. Additionally, propionate has been shown to inhibit the activity of 3-hydroxy-3-methyl-glutaryl-CoA reductase, thereby reducing endogenous cholesterol synthesis (Zhang et al., 2023; Louis and Flint, 2017). Butyrate is predominantly localized to the colon and cecum and is chiefly produced by members of the Firmicutes phylum. Most of the butyrate is absorbed and used by colonic epithelial cells, serving as their primary energy source. Only a small proportion of butyrate reaches the systemic circulation through the portal vein (Zhang et al., 2023; Louis and Flint, 2017; Soto-Martin et al., 2020).

## 3 HLAP induces gut dysbiosis and alterations in SCFAs

### 3.1 Gut dysbiosis in HLAP patients

The gastrointestinal microbiome, consisting of over 1,000 bacterial species, has been reported to contain a gene pool approximately 100 times larger than that of the human genome. More than 99% of these bacteria are classified into five predominant phyla: Firmicutes, Bacteroidetes, Proteobacteria, Verrucomicrobia, and Actinobacteria (Lupu et al., 2023). With the rapid advancement of microbiological research, increasing attention has been directed toward the role of gut dysbiosis in pancreatic diseases. In patients with AP, gut dysbiosis has been observed in comparison to healthy controls, characterized by an elevated abundance of opportunistic pathogens and a reduction in beneficial bacteria such as Firmicutes and Actinobacteria. Stratified analyses of AP cases have demonstrated that the composition of the gut microbiota varies according to disease severity, indicating a contributory role of intestinal microbes in the pathogenesis and progression of AP (Zhang et al., 2018; van den Berg et al., 2021; Yu et al., 2021; Yu et al., 2020).

Further investigations have indicated distinct gut microbial profiles in patients with HLAP compared to other forms of AP. In HLAP, more substantial reductions in both microbial abundance and diversity have been reported when compared to non-HLAP cases (Hu et al., 2021). These changes are primarily characterized by an increased prevalence of *Enterococcus* and *Escherichia*, accompanied by decreased levels of *Bacteroides* and *Faecalibacterium*. Correlation analyses have indicated a negative association between the abundance of *Faecalibacterium* and *Bacteroides* and the severity of disease, indicating a potential pathophysiological relationship between gut microbiota composition and HLAP (Li et al., 2023).

### 3.2 Gut dysbiosis leads to alterations in SCFAs

A bidirectional relationship has been identified between the gut microbiota, their metabolites, and the host. Among microbial metabolites, SCFAs represent the most abundant group in the gastrointestinal tract. As the principal end products of bacterial fermentation, SCFAs have been recognized as a key component in mediating host–microbiota interactions (Gentile and Weir, 2018). The primary SCFA-producing microorganisms are anaerobic bacteria, including genera such as *Bacteroides*, *Bifidobacterium*, *Clostridium*, and *Streptococcus* (Marchesi et al., 2016). These bacteria predominantly secrete acetic and lactic acids, which reduce intestinal pH and inhibit the proliferation of pathogenic microbes. In addition, they are known to degrade cellulose effectively, enabling the fermentation of dietary fiber and the subsequent production of SCFAs (Zhu et al., 2019).

In the context of HLAP, a decline in the abundance of these commensal intestinal bacteria has been usually observed, accompanied by an increase in opportunistic pathogens (Li et al., 2023). The reduction in SCFA-producing bacteria tends to become more pronounced with disease progression. Zhu et al. reported significantly lower levels of *Bacteroides*, *Alloprevotella*, *Blautia*, and *Gemella* in patients with severe acute pancreatitis (SAP) compared to those with mild and moderately severe (MSAP) forms (Zhu et al., 2019). These microbial taxa have previously facilitated dietary fiber fermentation and SCFA production (Chen et al., 2018; Liu et al., 2018; Kellingray et al., 2018). Furthermore, *Eubacterium hallii*, a known butyrate-producing bacterium, was identified by Yu et al. as one of the most significantly reduced genera in both MSAP and SAP patients (Yu et al., 2020). The reduction or disruption of these beneficial bacterial populations has been identified as a primary contributor to the decreased production of SCFAs in patients with HLAP.

## 4 Role of SCFAs in HLAP

### 4.1 SCFAs maintain gut homeostasis

Acute intestinal dysfunction is frequently observed as a complication in patients with HLAP and is strongly associated with unfavorable clinical outcomes (Ding et al., 2020). As SCFAs are primarily synthesized within the intestine, most current research on their mechanisms of action has focused on their role in maintaining gut homeostasis. Gut homeostasis refers to a dynamic and integrated equilibrium involving gut microbiota, intestinal epithelial barrier, and mucosal immune barrier. The protective effects of SCFAs can be broadly classified according to their influence on these three components.

First, SCFAs contribute to the correction of gut dysbiosis. *Clostridium butyricum*, an anaerobic bacterium capable of fermenting dietary fiber to produce SCFAs increases the abundance and diversity of intestinal microbiota when administered orally in HLAP rat models. This intervention promoted the proliferation of beneficial bacteria such as *Lactobacillus*, *Coprococcus*, and *Allobaculum*, while reducing the levels of pathogenic bacteria (Zhao et al., 2020). Direct supplementation with butyrate enhances the relative abundance of SCFA-producing bacteria within the intestinal tract (Gao et al., 2021; Xiong et al., 2022).

In addition, SCFAs serve as a primary energy source for intestinal epithelial cells and exert direct effects in maintaining the structural integrity of the intestinal barrier (Xiao et al., 2022). In rat models of SAP, treatment with butyrate resulted in decreased pathological severity scores of intestinal injury and reduced plasma concentrations of inflammatory markers. When compared to untreated controls, increased expression of tight junction proteins such as zonula occludens-1, claudin-1, and occludin were observed, along with decreased expression of claudin-2 and matrix metallopeptidase-9, indicating repair of the intestinal mucosal barrier (Zhao et al., 2020). Butyrate restores goblet cells responsible for mucin secretion, thereby contributing to mucosal protection (Gao et al., 2021).

Regarding the mucosal immune barrier, SCFAs modulate both colonic epithelial cells and immune cells to exert anti-inflammatory effects. Pretreatment with butyrate has been associated with attenuation of intestinal inflammation and injury through the suppression of pro-inflammatory cytokines such as tumor necrosis factor-α (TNF-α) and interleukin-6 (IL-6). Findings from immunofluorescence staining and flow cytometry analyses have further demonstrated increased expression of forkhead box protein 3 at both the mRNA and protein levels, supporting the role of butyrate in enhancing the proportion of regulatory T cells. These effects contribute to the prevention of excessive innate and adaptive immune responses and the preservation of gut homeostasis in SAP (Xiao et al., 2022; Fan et al., 2024).

### 4.2 SCFAs enhance lipid metabolism

Hyperlipidemia (HL) is a major contributing factor in the development of HLAP, with the incidence of HLAP having surpassed that of alcoholic pancreatitis, making it the second most common cause of AP (Chinese Pancreatic Surgery Association, and Chinese Society of Surgery, Chinese Medical Association, 2021). HL is a pathological condition resulting from disruptions in lipid metabolism caused by various internal and external factors. In this context, SCFAs have been identified as key regulators of host energy metabolism, influencing the balance of lipid degradation, fatty acid oxidation, and synthesis (Komaroff, 2017).

Diets high in fat and carbohydrates have been shown to reduce both the diversity and abundance of gut microbiota, particularly leading to a marked decline in SCFA-producing bacteria such as *Bacteroides* and *Bifidobacterium*. This microbial imbalance contributes to increased lipid accumulation and the onset of hyperlipidemia (Wang et al., 2020; Gallardo-Becerra et al., 2020; Ziętek et al., 2021). Experimental studies have demonstrated that continuous administration of fructooligosaccharides in mice significantly increases SCFA levels, lowers serum total cholesterol, triglycerides, and low-density lipoprotein levels induced by high-fat and high-carbohydrate diets. Furthermore, reductions in chronic inflammation, oxidative stress, and lipid deposition were observed, along with improved lipid profiles in the circulatory system (Schachter et al., 2018). Additional research has indicated that direct dietary supplementation with SCFAs promotes triglyceride hydrolysis, enhances FA oxidation, and facilitates the formation of brown adipose tissue, thereby reducing blood lipid levels in mice that were fed a high-fat diet (Lu et al., 2016). In summary, direct or indirect supplementation of SCFAs can significantly improve lipid metabolism. Previous studies have indicated that in severe hyperlipidemia, a large amount of lipoproteins and chylomicrons can lead to elevated plasma viscosity, which is believed to hinder blood flow in pancreatic tissue, leading to ischemia and tissue injury, and ultimately, acute pancreatitis (Adiamah et al., 2018). Furthermore, the easy availability of pro-inflammatory free fatty acids (FFAs) in plasma and the potential accumulation of FFAs in pancreatic tissue can contribute to the exacerbation of disease progression (Hansen et al., 2019; Hansen et al., 2023). Therefore, SCFA may prevent the onset and progression of HLAP by lowering blood lipid levels.

The mechanisms by which SCFAs regulate hyperlipidemia remain under active investigation. SCFAs have been found to serve dual roles in lipid metabolism, acting both as metabolic substrates through conversion into acetyl coenzyme A and as signaling molecules. SCFAs have been shown to activate the 5′-AMP-activated protein kinase (AMPK) signaling pathway, leading to increased expression of hormone-sensitive lipase (HSL) and adipose triglyceride lipase, thereby enhancing lipolysis (Tang et al., 2020). In addition, SCFAs downregulate the expression of peroxisome proliferator-activated receptor-γ and increase the expression of mitochondrial uncoupling protein 2 by activating AMPK and elevating the AMP/ATP ratio. These changes collectively contribute to enhanced FA oxidation in hepatic and adipose tissues (den Besten et al., 2015).

Further findings suggest that SCFAs inhibit peroxisome proliferator-activated receptor-α while concurrently activating AMPK and extracellular signal-regulated kinase 1/2 pathways, resulting in suppression of FA synthesis and promotion of FA catabolism and oxidation, thereby reducing lipid accumulation (Liu et al., 2019). Additionally, SCFAs impair ATP synthesis via activation of uncoupling proteins, leading to increased thermogenesis and lipid metabolic expenditure, thus improving overall lipid metabolism (He et al., 2020).

### 4.3 SCFAs modulate the immunoinflammatory process

The pathogenesis of HLAP is multifactorial, with several mechanisms contributing simultaneously and often intersecting. Among these, the inflammatory response plays a central role in both the initiation and progression of the disease. In the early stages of HLAP, pancreatic inflammation initiated a cytokine cascade that manifested clinically as SIRS (Methods in Medicine CAM, 2023). The excessive activation and amplification of inflammatory pathways represent key factors in HLAP development, making the prevention or timely interruption of SIRS essential for early disease management.

SCFAs have been identified as potential mediators of the regulatory effects of gut microbiota on both intestinal and systemic inflammatory responses. SCFAs not only exert local effects in the gut but also modulate immune cell function and regulate systemic inflammation via multiple inflammatory signaling pathways. According to evidence, a reduction in butyrate-producing bacterial strains disrupts SCFA synthesis and metabolism, thereby exacerbating HLAP progression and contributing to altered gut metabolic profiles. Butyrate exerts anti-inflammatory effects through inhibition of histone deacetylase 1 (HDAC1) and modulation of the signal transducer and activator of transcription 1 (STAT1)/AP1-NLRP3 signaling pathway (van den Berg et al., 2021). Additionally, *Parabacteroides* have been reported to produce acetate that alleviates heparanase-aggravated acute pancreatitis by reducing neutrophil infiltration (Lei et al., 2021). *Bifidobacterium* and its metabolite lactic acid suppress systemic inflammation and attenuate acute pancreatitis by modulating the Toll-like receptor 4 (TLR4)/MyD88 and NLRP3/Caspase-1 signaling pathways (Li et al., 2022). Furthermore, butyrate has been shown to reduce pancreatic injury in acute pancreatitis by downregulating inflammatory mediators and inhibiting activation of the NLRP3 inflammasome (Pan et al., 2019).

SCFAs exert diverse immunomodulatory effects on various immune and inflammatory cells. These metabolites have been shown to influence leukocyte function by promoting leukocyte migration and suppressing the production of pro-inflammatory cytokines (Vinolo et al., 2009; Park et al., 2007). In addition, SCFAs induce apoptosis in lymphocytes, macrophages, and neutrophils (Kurita-Ochiai et al., 2001; Bailón et al., 2010; Ramos et al., 2002; Aoyama et al., 2010). The mechanisms underlying SCFA activity are complex, with two primary pathways extensively studied: inhibition of HDAC activity to produce epigenetic effects and activation of GPCRs to initiate signal transduction.

SCFAs exhibit anti-inflammatory properties by inhibiting the release of pro-inflammatory mediators such as interleukin-1β (IL-1β), TNF-α, IL-6, and nitric oxide, while promoting the expression of the anti-inflammatory cytokine interleukin-10 (IL-10) (Ferreira et al., 2014; Cox et al., 2009). Butyrate inhibits the activation of nuclear factor kappa B (NF-κB) and STAT1 contributing to its anti-inflammatory effects (Belizário et al., 2018). Moreover, butyrate activates PPAR-γ, which is abundantly expressed in colonic epithelial cells, and suppresses interferon-γ signaling (Liu et al., 2018). As inhibitors of HDAC, SCFAs also influence gene expression by promoting protein hyperacetylation, facilitating chromatin remodeling, and modulating transcriptional activity, ultimately leading to cell cycle arrest and apoptosis (Chen et al., 2019). Collectively, these findings support the immunoregulatory potential of SCFAs in maintaining a balanced inflammatory response.

### 4.4 SCFAs protect organ function

In recent years, the incidence of HLAP has continued to rise, accompanied by a trend toward increased clinical severity and a higher prevalence of complications, including ARDS, AKI, and MODS (Li et al., 2018). SCFAs prevent or alleviate organ failure through the restoration of intestinal barrier integrity and inhibition of systemic inflammatory responses, with their effects extending to organs such as the lungs and kidneys.

Acute lung injury and ARDS are observed in approximately one-third of patients with SAP. The underlying pathogenesis of lung injury involves increased pulmonary microvascular permeability, resulting in the accumulation of protein-rich exudates within the alveolar spaces and the formation of hyaline membranes (Shields et al., 2002; Yehia Kamel et al., 2023). SCFAs produced by gut microbiota have been shown to reduce the expression of pro-inflammatory cytokines and reactive oxygen species, lower immune cell infiltration, and attenuate lipopolysaccharide-induced microvascular permeability and histological lung damage. These effects are mediated through the inhibition of high-mobility group box 1 protein release and NF-κB activation (Li et al., 2018; Verma et al., 2024). In hypoxic models, administration of butyrate reduces macrophage accumulation in alveolar and interstitial tissues, prevents hypoxia-induced pulmonary vascular edema and leakage, and upregulates tight junction protein expression in pulmonary microvascular endothelial cells (Karoor et al., 2021). In patients with acute pancreatitis and ARDS, an increased abundance of Proteobacteria, Enterobacteriaceae, *Escherichia-Shigella*, and *Klebsiella pneumoniae*, along with a decreased abundance of *Bifidobacterium*, has been reported in comparison to those without ARDS (Hu et al., 2023). These findings indicate that gut microbiota and SCFAs may play a key role in the development of pancreatitis-associated lung injury.

AKI is another common complication of SAP, with pathogenic mechanisms involving increased vascular permeability, hypovolemia, inflammatory responses, vasoconstriction, intravascular coagulation, and direct nephrotoxic damage (Nassar and Qunibi, 2019). SCFAs have demonstrated anti-inflammatory and immunomodulatory effects in AKI, contributing to improved renal function (Andrade-Oliveira et al., 2015). SCFA therapy has been shown to reduce pro-inflammatory cytokine and chemokine levels in renal tissue and serum by downregulating TLR4 mRNA expression and inhibiting NF-κB pathway activation. Concurrently, reductions in apoptotic cell counts in renal tissue and increased proliferation of renal epithelial cells have been observed, facilitating tissue repair (Andrade-Oliveira et al., 2015; Huang et al., 2017). Additional studies have reported that high-fiber diets confer similar protective effects in AKI. Dietary fiber improves AKI-associated gut dysbiosis by promoting the growth of SCFA-producing bacteria such as *Bifidobacterium* and *Prevotella*. Inhibition of renal HDAC activity has also been observed in mice that have been fed a high-fiber diet (Liu et al., 2021).

Evidence shows that SCFAs contribute to the mitigation of pancreatic injury in AP (Sun et al., 2015). In murine models of AP, pretreatment with sodium butyrate has been shown to reduce macrophage and neutrophil infiltration into pancreatic tissue and decrease pro-inflammatory cytokine levels in the intestine. These effects may be mediated via inhibition of HDAC1 in the pancreas or activation of G protein-coupled receptor 109A in the colon, leading to suppression of NLRP3 inflammasome activation (Pan et al., 2019). Another study demonstrated that sodium butyrate supplementation significantly reduced the proportions of neutrophils, macrophages, and M2-type macrophages in pancreatic tissue, along with decreased expression of IL-1β, TNF-α, and C-X-C motif chemokine ligand 1 (Xiong et al., 2022).

**Figure 2:**
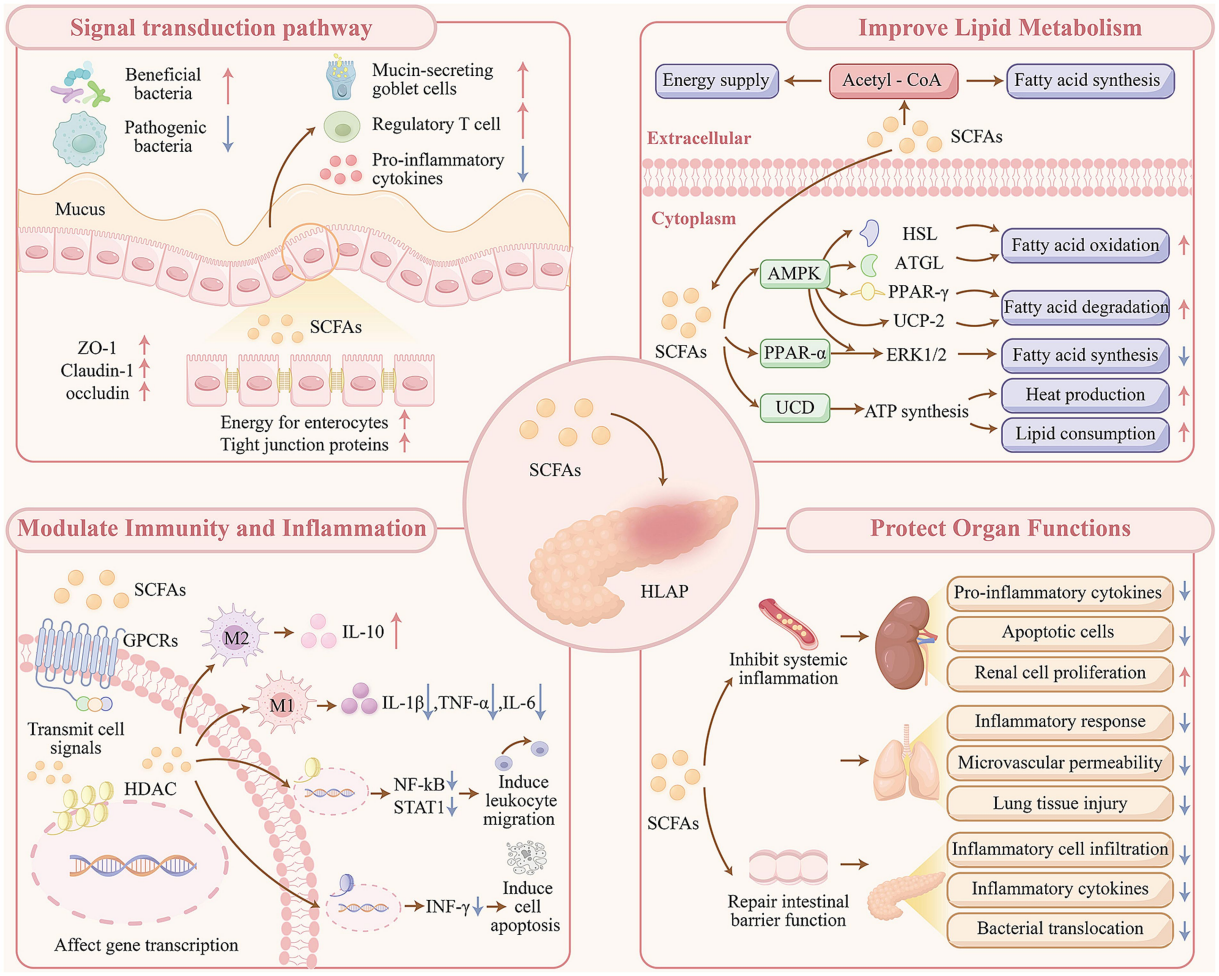
Mechanistic pathways of SCFA-mediated modulation in HLAP progression.

The persistence of systemic inflammation or local pancreatic complications markedly increases the risk of infectious events in patients with acute pancreatitis, including pancreatic abscesses, bloodstream infections, and pulmonary infections (Besselink et al., 2009). Given the ability of SCFAs to protect intestinal barrier function and reduce pancreatic injury, their supplementation as part of early enteral nutrition may offer a promising strategy for lowering the incidence of systemic infectious complications in individuals with HLAP (see Figure 2).

## 5 Potential of SCFAs in HLAP therapy

Patients with HLAP frequently exhibit gut dysbiosis, particularly marked by a reduction in SCFA-producing bacteria, which may result in significantly decreased SCFA concentrations within the intestinal tract. As the decline in SCFAs is considered a key contributor to the pathogenesis and progression of HLAP, both direct and indirect supplementation of SCFAs may represent promising therapeutic strategies. The main approaches are outlined as follows:

1. Direct supplementation of SCFAs: Preclinical studies have indicated that oral or systemic administration of butyrate in HLAP mouse models significantly reduces mortality and the translocation of *Escherichia coli*, while also reversing gut dysbiosis (van den Berg et al., 2021). Clinical trials have demonstrated beneficial effects of SCFA therapy in intestinal disorders such as ulcerative colitis and radiation proctitis (Hamer et al., 2010; Firoozi et al., 2024; Vernia et al., 2000). Although no clinical trials have evaluated SCFA efficacy in patients with HLAP as yet, existing evidence indicates that incorporation of SCFAs into early enteral nutrition may be beneficial, particularly for those with concurrent intestinal dysfunction. This method may offer a safer alternative to probiotic supplementation, which carries a risk of bacteremia. However, maintaining physiologically relevant SCFA concentrations in the gut and plasma presents a major challenge due to their rapid systemic metabolism and utilization as energy substrates (Müller et al., 2019; Shubitowski et al., 2019). Therefore, the development of optimal administration routes and efficient delivery systems remains critical for advancing clinical application.
2. Increasing dietary fiber intake: SCFAs are primarily produced through the fermentation of dietary fiber by gut microbiota. In patients with HLAP, reductions in microbial abundance and SCFA concentrations reflect suppressed colonic fermentation and intestinal dysbiosis. A randomized controlled trial (RCT) reported that the addition of dietary fiber to early enteral nutrition in patients with SAP resulted in shortened hospital stays, reduced acute-phase responses, and decreased complication rates (Karakan et al., 2007). These outcomes are attributed to improved intestinal function and increased SCFA production. However, the effectiveness of dietary fiber supplementation may vary depending on individual factors, including the abundance of SCFA-producing bacteria and intestinal motility, leading to inter-individual variability in treatment response.
3. Probiotic supplementation: Probiotics and their metabolites contribute to intestinal homeostasis by inhibiting pathogenic organisms (Colautti et al., 2022), enhancing intestinal barrier integrity (Engevik et al., 2019; Huang et al., 2020), and modulating immune responses (La Fata et al., 2018). Several RCTs have reported that probiotics reduce the incidence of complications and shorten hospitalization in patients with SAP, while also preventing organ dysfunction (Oláh et al., 2002; Oláh et al., 2007; Cui et al., 2013; Liu et al., 2023). However, meta-analyses have not found significant differences in the incidence of SIRS, infected pancreatic necrosis, surgical interventions, sepsis, or mortality between probiotic and control groups (Yu et al., 2021). Potential risks such as antibiotic resistance, allergic reactions, infections, and sepsis should be considered before clinical use of probiotics (Stapleton and McClave, 2009). As such, the therapeutic role of probiotics in HLAP remains controversial.
4. Fecal microbial transplantation (FMT): FMT is an emerging therapeutic modality that aims to restore the intestinal microbiota directly. It has been recommended for use in conditions such as Clostridioides difficile infection (Cammarota et al., 2017), ulcerative colitis (Paramsothy et al., 2019), irritable bowel syndrome (Xu et al., 2019), and hepatic encephalopathy (Bajaj et al., 2017). However, its application in HLAP remains poorly defined. Limited preclinical studies have evaluated FMT in mouse models of AP, with some reporting improvements in gut dysbiosis and disease severity following transplantation of normal microbiota (Liu et al., 2023). Relevant studies have also found that during experimental AP induction, mice fed a western diet (WD) exhibited elevated blood lipids, enhanced bacterial dissemination, aggravated systemic inflammatory responses, and increased mortality compared with those fed a standard diet (SD). These outcomes were correlated with a reduction in gut microbial diversity and a decline in the abundance of SCFA-producing bacteria (van den Berg et al., 2021). Therefore, we hypothesize that FMT may regulate gut microbiota, increase the production of SCFAs, and ameliorate the prognosis of HLAP.

However, surprisingly, the team found that FMT treatment unexpectedly increased the mortality rate of mice, the bacterial culture positivity rate and total colony-forming units (CFU) of pancreatic tissue. These phenomenon may be attributed to altered intestinal permeability, bacterial translocation (Fishman et al., 2014; Liang et al., 2014), and/or pancreatic contamination caused by bacterial reflux into the pancreatic duct during the experiment (Pushalkar et al., 2018). Given the limited and controversial results of animal experiments, along with the multitude of factors influencing FMT, such as donor selection, host factors, bacterial volume, frequency, and administration methods (Porcari et al., 2023), further specialized research is needed on the efficacy and safety of FMT in HLAP treatment, which requires large-scale and well-designed studies.

## 6 Summary and future prospects

HLAP is a common cause of acute abdominal pain that originates from pancreatic inflammation; however, the intestinal tract serves as an amplifier of the disease process by intensifying and potentially perpetuating systemic inflammatory responses. Therefore, preserving the integrity of intestinal function is considered essential in the management of inflammation during HLAP. Accumulating evidence indicates that patients with HLAP frequently exhibit gut dysbiosis, characterized by reduced microbial diversity and a reduced abundance of SCFA-producing bacteria. SCFAs, as key metabolites derived from the gut microbiota play a vital role in maintaining intestinal homeostasis, regulating lipid metabolism, modulating immune responses, and protecting organ function.

As a result, increasing attention has been directed toward understanding the pathogenesis of HLAP through the lens of gut microbiota and their metabolic products. Given the wide-ranging beneficial effects of SCFAs, strategies aimed at increasing SCFA levels are considered promising for enhancing intestinal protection in HLAP. These strategies include direct SCFA supplementation or indirect methods such as increasing dietary fiber intake, probiotic administration, and FMT. However, current interventions targeting gut microbiota and their metabolites in HLAP remain largely limited to preclinical models and mechanistic studies. Further research involving rigorously designed clinical trials is required to assess optimal methods of administration and the clinical efficacy of SCFA-based therapies. In addition, due to the complex and aggressive nature of HLAP, the safety profile of SCFA supplementation must be carefully evaluated.

## Glossary

HLAP: Hypertriglyceridemia-associated acute pancreatitis
SCFAs: Short-chain fatty acids
AP: Acute pancreatitis
ARDS: Acute respiratory distress syndrome
AKI: Acute kidney injury
MODS: Multiple organ dysfunction syndrome
SIRS: Systemic inflammatory response syndrome
HDAC: Histone deacetylase
GPCRs: G protein coupled receptor
HMG-CoA: 3-hydroxy-3-methyl-glutaryl-CoA
ZO-1: Zonula occludens-1
MMP9: Matrix metallopeptidases-9
TNF-α: Tumor necrosis factor-α
IL-6: Interleukin-6
HL: Hyperlipidaemia
TC: Total cholesterol
TG: Triglycerides
LDL: Low-density lipoprotein
Acetyl-CoA: Acetyl coenzyme A
AMPK: 5-Monophosphate-activated protein kinase
HSL: Hormone-sensitive lipase
ATGL: Adipose triglyceride lipase
PPARγ: Peroxisome proliferator-activated receptor-γ
AMP: Adenosine monophosphate
ATP: Adenosine triphosphate
FA: Fatty acid
STAT1: Signal transducer and activator of transcription 1
AP1: Activator protein 1
NLRP3: Nucleotide-binding oligomerization domain-like receptor protein 3
TLR4: Toll-like receptor 4
MyD88: Myeloid differentiation primary response 88
Caspase1: Cysteine-aspartic protease 1
IL-1β: Interleukin-1β
IL-10: Interleukin-10
NO: Nitric oxide
NF-κB: Nuclear factor-kappa B
IFN-γ: Interferon-gamma
HMGB1: High-mobility group box 1 protein
LPS: Lipopolysaccharide
GPR109A: G protein-coupled receptor 109A
CXCL1: C-X-C motif chemokine ligand 1
UCP-2: Uncoupling protein-2
PPAR-α: Proliferator-activated receptor-α
ERK1/2: Extracellular-signal-regulated kinase 1/2
UCP: Uncoupling protein
ALI: Acute lung injury
FMT: Fecal microbial transplantation

## References

[ref1] Adiamah A. Psaltis E. Crook M. Lobo D. N. (2018). A systematic review of the epidemiology, pathophysiology and current management of hyperlipidaemic pancreatitis. Clin. Nutr. 37, 1810–1822. doi: 10.1016/j.clnu.2017.09.028, PMID: 29056284

[ref2] Andrade-Oliveira V. Amano M. T. Correa-Costa M. Castoldi A. Felizardo R. J. de Almeida D. C.. (2015). Gut Bacteria products prevent AKI induced by ischemia-reperfusion. J. Am. Soc. Nephrol. 26, 1877–1888. doi: 10.1681/ASN.2014030288 25589612 PMC4520159

[ref3] Aoyama M. Kotani J. Usami M. (2010). Butyrate and propionate induced activated or non-activated neutrophil apoptosis via HDAC inhibitor activity but without activating GPR-41/GPR-43 pathways. Nutrition 26, 653–661. doi: 10.1016/j.nut.2009.07.006, PMID: 20004081

[ref4] Bailón E. Cueto-Sola M. Utrilla P. Rodríguez-Cabezas M. E. Garrido-Mesa N. Zarzuelo A.. (2010). Butyrate *in vitro* immune-modulatory effects might be mediated through a proliferation-related induction of apoptosis. Immunobiology 215, 863–873. doi: 10.1016/j.imbio.2010.01.001, PMID: 20149475

[ref5] Bajaj J. S. Kassam Z. Fagan A. Gavis E. A. Liu E. Cox I. J.. (2017). Fecal microbiota transplant from a rational stool donor improves hepatic encephalopathy: a randomized clinical trial. Hepatology 66, 1727–1738. doi: 10.1002/hep.29306 28586116 PMC6102730

[ref6] Belizário J. E. Faintuch J. Garay-Malpartida M. (2018). Gut microbiome Dysbiosis and Immunometabolism: new Frontiers for treatment of metabolic diseases. Mediat. Inflamm. 9:2037838. doi: 10.1155/2018/2037838, PMID: 30622429 PMC6304917

[ref7] Besselink M. G. van Santvoort H. C. Boermeester M. A. Nieuwenhuijs V. B. van Goor H. Dejong C. H.. (2009). Timing and impact of infections in acute pancreatitis. Br. J. Surg. 96, 267–273. doi: 10.1002/bjs.6447, PMID: 19125434

[ref8] Cammarota G. Ianiro G. Tilg H. Rajilić-Stojanović M. Kump P. Satokari R.. (2017). European FMT working group. European consensus conference on faecal microbiota transplantation in clinical practice. Gut 66, 569–580. doi: 10.1136/gutjnl-2016-313017 28087657 PMC5529972

[ref9] Chen J. Kang B. Jiang Q. Han M. Zhao Y. Long L.. (2018). Alpha-ketoglutarate in low-protein diets for growing pigs: effects on Cecal microbial communities and parameters of microbial metabolism. Front. Microbiol. 31:1057. doi: 10.3389/fmicb.2018.01057 PMC5991137 29904374

[ref10] Chen J. Zhao K. N. Vitetta L. (2019). Effects of intestinal microbial^−^elaborated butyrate on oncogenic signaling pathways. Nutrients 11:1026. doi: 10.3390/nu11051026, PMID: 31067776 PMC6566851

[ref11] Chinese Pancreatic Surgery Association, and Chinese Society of Surgery, Chinese Medical Association (2021). Guidelines for diagnosis and treatment of acute pancreatitis in China (2021). Zhonghua Wai Ke Za Zhi 59, 578–587. Chinese. doi: 10.3760/cma.j.cn112139-20210416-00172, PMID: 34256457

[ref12] Colautti A. Orecchia E. Comi G. Iacumin L. (2022). Lactobacilli, a weapon to counteract pathogens through the inhibition of their virulence factors. J. Bacteriol. 204:e0027222. doi: 10.1128/jb.00272-22 36286515 PMC9664955

[ref13] Cox M. A. Jackson J. Stanton M. Rojas-Triana A. Bober L. Laverty M.. (2009). Short-chain fatty acids act as antiinflammatory mediators by regulating prostaglandin E(2) and cytokines. World J. Gastroenterol. 15, 5549–5557. doi: 10.3748/wjg.15.5549, PMID: 19938193 PMC2785057

[ref14] Cui L. H. Wang X. H. Peng L. H. Yu L. Yang Y. S. (2013). The effects of early enteral nutrition with addition of probiotics on the prognosis of patients suffering from severe acute pancreatitis. Zhonghua Wei Zhong Bing Ji Jiu Yi Xue 25, 224–228. Chinese. doi: 10.3760/cma.j.issn.2095-4352.2013.04.011, PMID: 23660099

[ref15] den Besten G. Bleeker A. Gerding A. van Eunen K. Havinga R. van Dijk T. H.. (2015). Short-chain fatty acids protect against high-fat diet-induced obesity via a PPARγ-dependent switch from lipogenesis to fat oxidation. Diabetes 64, 2398–2408. doi: 10.2337/db14-1213, PMID: 25695945

[ref16] Ding L. Chen H. Y. Wang J. Y. Xiong H. F. He W. H. Xia L.. (2020). Severity of acute gastrointestinal injury grade is a good predictor of mortality in critically ill patients with acute pancreatitis. World J. Gastroenterol. 26, 514–523. doi: 10.3748/wjg.v26.i5.514, PMID: 32089627 PMC7015716

[ref17] Engevik M. A. Luk B. Chang-Graham A. L. Hall A. Herrmann B. Ruan W.. (2019). *Bifidobacterium dentium* fortifies the intestinal mucus layer via autophagy and calcium signaling pathways. MBio 10:e01087-19. doi: 10.1128/mBio.01087-19, PMID: 31213556 PMC6581858

[ref18] Facchin S. Bertin L. Bonazzi E. Lorenzon G. De Barba C. Barberio B.. (2024). Short-chain fatty acids and human health: from metabolic pathways to current therapeutic implications. Life (Basel) 14:559. doi: 10.3390/life14050559, PMID: 38792581 PMC11122327

[ref19] Fan J. N. Ho H. Chiang B. L. (2024). Characterization of novel CD8+ regulatory T cells and their modulatory effects in murine model of inflammatory bowel disease. Cell. Mol. Life Sci. 81:327. doi: 10.1007/s00018-024-05378-x, PMID: 39085655 PMC11335251

[ref20] Ferreira M. R. Muls A. Dearnaley D. P. Andreyev H. J. (2014). Microbiota and radiation-induced bowel toxicity: lessons from inflammatory bowel disease for the radiation oncologist. Lancet Oncol. 15, e139–e147. doi: 10.1016/S1470-2045(13)70504-7, PMID: 24599929

[ref21] Firoozi D. Masoumi S. J. Mohammad-Kazem Hosseini Asl S. Labbe A. Razeghian-Jahromi I. Fararouei M.. (2024). Effects of short-chain fatty acid-butyrate supplementation on expression of circadian-clock genes, sleep quality, and inflammation in patients with active ulcerative colitis: a double-blind randomized controlled trial. Lipids Health Dis. 23:216. doi: 10.1186/s12944-024-02203-z, PMID: 39003477 PMC11245831

[ref22] Fishman J. E. Levy G. Alli V. Zheng X. Mole D. J. Deitch E. A. (2014). The intestinal mucus layer is a critical component of the gut barrier that is damaged during acute pancreatitis. Shock 42, 264–270. doi: 10.1097/SHK.0000000000000209, PMID: 24978882 PMC4134397

[ref23] Gallardo-Becerra L. Cornejo-Granados F. García-López R. Valdez-Lara A. Bikel S. Canizales-Quinteros S.. (2020). Metatranscriptomic analysis to define the Secrebiome, and 16S rRNA profiling of the gut microbiome in obesity and metabolic syndrome of Mexican children. Microb. Cell Factories 19:61. doi: 10.1186/s12934-020-01319-y, PMID: 32143621 PMC7060530

[ref24] Gao Y. Davis B. Zhu W. Zheng N. Meng D. Walker W. A. (2021). Short-chain fatty acid butyrate, a breast milk metabolite, enhances immature intestinal barrier function genes in response to inflammation *in vitro* and *in vivo*. Am. J. Physiol. Gastrointest. Liver Physiol. 320, G521–G530. doi: 10.1152/ajpgi.00279.2020 33085904 PMC8238162

[ref25] Gentile C. L. Weir T. L. (2018). The gut microbiota at the intersection of diet and human health. Science 362, 776–780. doi: 10.1126/science.aau5812, PMID: 30442802 PMC13264711

[ref26] Hamer H. M. Jonkers D. M. Vanhoutvin S. A. Troost F. J. Rijkers G. de Bruïne A.. (2010). Effect of butyrate enemas on inflammation and antioxidant status in the colonic mucosa of patients with ulcerative colitis in remission. Clin. Nutr. 29, 738–744. doi: 10.1016/j.clnu.2010.04.002, PMID: 20471725

[ref27] Hansen S. E. J. Madsen C. M. Varbo A. Nordestgaard B. G. (2019). Low-grade inflammation in the association between mild-to-moderate hypertriglyceridemia and risk of acute pancreatitis: a study of more than 115000 individuals from the general population. Clin. Chem. 65, 321–332. doi: 10.1373/clinchem.2018.294926, PMID: 30518661

[ref28] Hansen S. E. J. Varbo A. Nordestgaard B. G. Langsted A. (2023). Hypertriglyceridemia-associated pancreatitis: new concepts and potential mechanisms. Clin. Chem. 69, 1132–1144. doi: 10.1093/clinchem/hvad094, PMID: 37530032

[ref29] He J. Zhang P. Shen L. Niu L. Tan Y. Chen L.. (2020). Short-chain fatty acids and their association with signalling pathways in inflammation, glucose and lipid metabolism. Int. J. Mol. Sci. 21:6356. doi: 10.3390/ijms21176356, PMID: 32887215 PMC7503625

[ref30] Hu X. Gong L. Zhou R. Han Z. Ji L. Zhang Y.. (2021). Variations in gut microbiome are associated with prognosis of hypertriglyceridemia-associated acute pancreatitis. Biomolecules 11:695. doi: 10.3390/biom11050695, PMID: 34066441 PMC8148198

[ref31] Hu X. Han Z. Zhou R. Su W. Gong L. Yang Z.. (2023). Altered gut microbiota in the early stage of acute pancreatitis were related to the occurrence of acute respiratory distress syndrome. Front. Cell. Infect. Microbiol. 6:1127369. doi: 10.3389/fcimb.2023.1127369, PMID: 36949815 PMC10025409

[ref32] Huang F. C. Lu Y. T. Liao Y. H. (2020). Beneficial effect of probiotics on *Pseudomonas aeruginosa*-infected intestinal epithelial cells through inflammatory IL-8 and antimicrobial peptide human beta-defensin-2 modulation. Innate Immun. 26, 592–600. doi: 10.1177/1753425920959410 32988256 PMC7556188

[ref33] Huang W. Zhou L. Guo H. Xu Y. Xu Y. (2017). The role of short-chain fatty acids in kidney injury induced by gut-derived inflammatory response. Metabolism 68, 20–30. doi: 10.1016/j.metabol.2016.11.006, PMID: 28183450

[ref34] Karakan T. Ergun M. Dogan I. Cindoruk M. Unal S. (2007). Comparison of early enteral nutrition in severe acute pancreatitis with prebiotic fiber supplementation versus standard enteral solution: a prospective randomized double-blind study. World J. Gastroenterol. 13, 2733–2737. doi: 10.3748/wjg.v13.i19.2733 17569144 PMC4147124

[ref35] Karoor V. Strassheim D. Sullivan T. Verin A. Umapathy N. S. Dempsey E. C.. (2021). The short-chain fatty acid butyrate attenuates pulmonary vascular remodeling and inflammation in hypoxia-induced pulmonary hypertension. Int. J. Mol. Sci. 22:9916. doi: 10.3390/ijms22189916, PMID: 34576081 PMC8467617

[ref36] Kellingray L. Gall G. L. Defernez M. Beales I. L. P. Franslem-Elumogo N. Narbad A. (2018). Microbial taxonomic and metabolic alterations during faecal microbiota transplantation to treat *Clostridium difficile* infection. J. Infect. 77, 107–118. doi: 10.1016/j.jinf.2018.04.012, PMID: 29746938

[ref37] Komaroff A. L. (2017). The microbiome and risk for obesity and diabetes. JAMA 317, 355–356. doi: 10.1001/jama.2016.20099, PMID: 28006047

[ref38] Kurita-Ochiai T. Ochiai K. Fukushima K. (2001). Butyric acid-induced T-cell apoptosis is mediated by caspase-8 and -9 activation in a Fas-independent manner. Clin. Diagn. Lab. Immunol. 8, 325–332. doi: 10.1128/CDLI.8.2.325-332.2001, PMID: 11238216 PMC96057

[ref39] La Fata G. Weber P. Mohajeri M. H. (2018). Probiotics and the gut immune system: indirect regulation. Probiotics Antimicrob. Proteins 10, 11–21. doi: 10.1007/s12602-017-9322-6, PMID: 28861741 PMC5801397

[ref40] Lei Y. Tang L. Liu S. Hu S. Wu L. Liu Y.. (2021). *Parabacteroides* produces acetate to alleviate heparanase-exacerbated acute pancreatitis through reducing neutrophil infiltration. Microbiome 9:115. doi: 10.1186/s40168-021-01065-2, PMID: 34016163 PMC8138927

[ref41] Li X. Y. He C. Zhu Y. Lu N. H. (2020). Role of gut microbiota on intestinal barrier function in acute pancreatitis. World J. Gastroenterol. 26, 2187–2193. doi: 10.3748/wjg.v26.i18.2187, PMID: 32476785 PMC7235204

[ref42] Li X. Ke L. Dong J. Ye B. Meng L. Mao W.. (2018). Significantly different clinical features between hypertriglyceridemia and biliary acute pancreatitis: a retrospective study of 730 patients from a tertiary center. BMC Gastroenterol. 18:89. doi: 10.1186/s12876-018-0821-z, PMID: 29914404 PMC6007076

[ref43] Li N. Liu X. X. Hong M. Huang X. Z. Chen H. Xu J. H.. (2018). Sodium butyrate alleviates LPS-induced acute lung injury in mice via inhibiting HMGB1 release. Int. Immunopharmacol. 56, 242–248. doi: 10.1016/j.intimp.2018.01.017, PMID: 29414658

[ref44] Li G. Liu L. Lu T. Sui Y. Zhang C. Wang Y.. (2023). Gut microbiota aggravates neutrophil extracellular traps-induced pancreatic injury in hypertriglyceridemic pancreatitis. Nat. Commun. 14:6179. doi: 10.1038/s41467-023-41950-y, PMID: 37794047 PMC10550972

[ref45] Li Z. Quan G. Jiang X. Yang Y. Ding X. Zhang D.. (2018). Effects of metabolites derived from gut microbiota and hosts on pathogens. Front. Cell. Infect. Microbiol. 14:314. doi: 10.3389/fcimb.2018.00314, PMID: 30276161 PMC6152485

[ref46] Li H. Xie J. Guo X. Yang G. Cai B. Liu J.. (2022). Bifidobacterium spp. and their metabolite lactate protect against acute pancreatitis via inhibition of pancreatic and systemic inflammatory responses. Gut Microbes 14:2127456. doi: 10.1080/19490976.2022.2127456, PMID: 36195972 PMC9542615

[ref47] Liang H. Y. Chen T. Wang T. Huang Z. Yan H. T. Tang L. J. (2014). Time course of intestinal barrier function injury in a sodium taurocholate-induced severe acute pancreatitis in rat model. J. Dig. Dis. 15, 386–393. doi: 10.1111/1751-2980.12148, PMID: 24690434

[ref48] Liu X. Cao J. N. Liu T. Zhong H. Liu M. Chang X. R.. (2023). Effect of herb-partitioned moxibustion on structure and functional prediction of gut microbiota in rats with irritable bowel syndrome with diarrhea. World J. Tradit. Chin. Med. 9, 141–149. doi: 10.4103/2311-8571.373586

[ref49] Liu L. Fu C. Li F. (2019). Acetate affects the process of lipid metabolism in rabbit liver, skeletal muscle and adipose tissue. Animals (Basel) 9:799. doi: 10.3390/ani9100799, PMID: 31615062 PMC6826666

[ref50] Liu Y. Li Y. J. Loh Y. W. Singer J. Zhu W. Macia L.. (2021). Fiber derived microbial metabolites prevent acute kidney injury through G-protein coupled receptors and HDAC inhibition. Front. Cell Dev. Biol. 8:648639. doi: 10.3389/fcell.2021.648639 PMC8060457 33898439

[ref51] Liu H. Wang J. He T. Becker S. Zhang G. Li D.. (2018). Butyrate: a double-edged sword for health? Adv. Nutr. 9, 21–29. doi: 10.1093/advances/nmx009, PMID: 29438462 PMC6333934

[ref52] Liu L. W. Xie Y. Li G. Q. Zhang T. Sui Y. H. Zhao Z. J.. (2023). Gut microbiota-derived nicotinamide mononucleotide alleviates acute pancreatitis by activating pancreatic SIRT3 signalling. Br. J. Pharmacol. 180, 647–666. doi: 10.1111/bph.15980, PMID: 36321732

[ref53] Liu J. Yue S. Yang Z. Feng W. Meng X. Wang A.. (2018). Oral hydroxysafflor yellow a reduces obesity in mice by modulating the gut microbiota and serum metabolism. Pharmacol. Res. 134, 40–50. doi: 10.1016/j.phrs.2018.05.012, PMID: 29787870

[ref54] Louis P. Flint H. J. (2017). Formation of propionate and butyrate by the human colonic microbiota. Environ. Microbiol. 19, 29–41. doi: 10.1111/1462-2920.13589, PMID: 27928878

[ref55] Lu Y. Fan C. Li P. Lu Y. Chang X. Qi K. (2016). Short chain fatty acids prevent high-fat-diet-induced obesity in mice by regulating G protein-coupled receptors and gut microbiota. Sci. Rep. 28:37589. doi: 10.1038/srep37589 PMC5124860 27892486

[ref56] Lupu V. V. Adam Raileanu A. Mihai C. M. Morariu I. D. Lupu A. Starcea I. M.. (2023). The implication of the gut microbiome in heart failure. Cells 12:1158. doi: 10.3390/cells12081158, PMID: 37190067 PMC10136760

[ref57] Marchesi J. R. Adams D. H. Fava F. Hermes G. D. Hirschfield G. M. Hold G.. (2016). The gut microbiota and host health: a new clinical frontier. Gut 65, 330–339. doi: 10.1136/gutjnl-2015-309990, PMID: 26338727 PMC4752653

[ref58] Methods in Medicine CAM (2023). Retracted: downregulation of miR-146a-5p promotes acute pancreatitis through activating the TLR9/NLRP3 signaling pathway by targeting TRAF6 *in vitro* rat model. Comput. Math. Methods Med. 6:9820687. doi: 10.1155/2023/9820687, PMID: 38094377 PMC10718954

[ref59] Müller M. Hernández M. A. G. Goossens G. H. Reijnders D. Holst J. J. Jocken J. W. E.. (2019). Circulating but not faecal short-chain fatty acids are related to insulin sensitivity, lipolysis and GLP-1 concentrations in humans. Sci. Rep. 9:12515. doi: 10.1038/s41598-019-48775-0, PMID: 31467327 PMC6715624

[ref60] Nassar T. I. Qunibi W. Y. (2019). AKI associated with acute pancreatitis. Clin. J. Am. Soc. Nephrol. 14, 1106–1115. doi: 10.2215/CJN.13191118 31118209 PMC6625613

[ref61] Niccolai E. Baldi S. Ricci F. Russo E. Nannini G. Menicatti M.. (2019). Evaluation and comparison of short chain fatty acids composition in gut diseases. World J. Gastroenterol. 25, 5543–5558. doi: 10.3748/wjg.v25.i36.5543, PMID: 31576099 PMC6767983

[ref62] Oláh A. Belágyi T. Issekutz A. Gamal M. E. Bengmark S. (2002). Randomized clinical trial of specific lactobacillus and fibre supplement to early enteral nutrition in patients with acute pancreatitis. Br. J. Surg. 89, 1103–1107. doi: 10.1046/j.1365-2168.2002.02189.x, PMID: 12190674

[ref63] Oláh A. Belágyi T. Pótó L. Romics L. Jr. Bengmark S. (2007). Synbiotic control of inflammation and infection in severe acute pancreatitis: a prospective, randomized, double blind study. Hepato-Gastroenterology 54, 590–594 17523328

[ref64] Pan X. Fang X. Wang F. Li H. Niu W. Liang W.. (2019). Butyrate ameliorates caerulein-induced acute pancreatitis and associated intestinal injury by tissue-specific mechanisms. Br. J. Pharmacol. 176, 4446–4461. doi: 10.1111/bph.14806 31347703 PMC6932943

[ref65] Paramsothy S. Nielsen S. Kamm M. A. Deshpande N. P. Faith J. J. Clemente J. C.. (2019). Specific bacteria and metabolites associated with response to fecal microbiota transplantation in patients with ulcerative colitis. Gastroenterology 156, 1440–1454.e2. doi: 10.1053/j.gastro.2018.12.001 30529583

[ref66] Park J. S. Lee E. J. Lee J. C. Kim W. K. Kim H. S. (2007). Anti-inflammatory effects of short chain fatty acids in IFN-gamma-stimulated RAW 264.7 murine macrophage cells: involvement of NF-kappaB and ERK signaling pathways. Int. Immunopharmacol. 7, 70–77. doi: 10.1016/j.intimp.2006.08.015, PMID: 17161819

[ref67] Porcari S. Benech N. Valles-Colomer M. Segata N. Gasbarrini A. Cammarota G.. (2023). Key determinants of success in fecal microbiota transplantation: from microbiome to clinic. Cell Host Microbe 31, 712–733. doi: 10.1016/j.chom.2023.03.020, PMID: 37167953

[ref68] Pouteau E. Nguyen P. Ballèvre O. Krempf M. (2003). Production rates and metabolism of short-chain fatty acids in the colon and whole body using stable isotopes. Proc. Nutr. Soc. 62, 87–93. doi: 10.1079/PNS2003208, PMID: 12740063

[ref69] Pushalkar S. Hundeyin M. Daley D. Zambirinis C. P. Kurz E. Mishra A.. (2018). The pancreatic cancer microbiome promotes oncogenesis by induction of innate and adaptive immune suppression. Cancer Discov. 8, 403–416. doi: 10.1158/2159-8290.CD-17-1134, PMID: 29567829 PMC6225783

[ref70] Ramos M. G. Rabelo F. L. Duarte T. Gazzinelli R. T. Alvarez-Leite J. I. (2002). Butyrate induces apoptosis in murine macrophages via caspase-3, but independent of autocrine synthesis of tumor necrosis factor and nitric oxide. Braz. J. Med. Biol. Res. 35, 161–173. doi: 10.1590/s0100-879x2002000200004, PMID: 11847519

[ref71] Rooks M. G. Garrett W. S. (2016). Gut microbiota, metabolites and host immunity. Nat. Rev. Immunol. 16, 341–352. doi: 10.1038/nri.2016.42, PMID: 27231050 PMC5541232

[ref72] Schachter J. Martel J. Lin C. S. Chang C. J. Wu T. R. Lu C. C.. (2018). Effects of obesity on depression: a role for inflammation and the gut microbiota. Brain Behav. Immun. 69, 1–8. doi: 10.1016/j.bbi.2017.08.026, PMID: 28888668

[ref73] Shields C. J. Winter D. C. Redmond H. P. (2002). Lung injury in acute pancreatitis: mechanisms, prevention, and therapy. Curr. Opin. Crit. Care 8, 158–163. doi: 10.1097/00075198-200204000-00012, PMID: 12386518

[ref74] Shubitowski T. B. Poll B. G. Natarajan N. Pluznick J. L. (2019). Short-chain fatty acid delivery: assessing exogenous administration of the microbiome metabolite acetate in mice. Physiol. Rep. 7:e14005. doi: 10.14814/phy2.14005, PMID: 30810289 PMC6391713

[ref75] Soto-Martin E. C. Warnke I. Farquharson F. M. Christodoulou M. Horgan G. Derrien M.. (2020). Vitamin biosynthesis by human gut butyrate-producing bacteria and cross-feeding in synthetic microbial communities. mBio 11:e00886-20. doi: 10.1128/mBio.00886-20, PMID: 32665271 PMC7360928

[ref76] Stapleton J. R. McClave S. A. (2009). Controversial results with use of probiotics in critical illness: contradictory findings from large multicenter trial. Curr. Gastroenterol. Rep. 11, 259–262. doi: 10.1007/s11894-009-0052-0, PMID: 19615300

[ref77] Sun J. Furio L. Mecheri R. van der Does A. M. Lundeberg E. Saveanu L.. (2015). Pancreatic β-cells limit autoimmune diabetes via an immunoregulatory antimicrobial peptide expressed under the influence of the gut microbiota. Immunity 43, 304–317. doi: 10.1016/j.immuni.2015.07.013 26253786

[ref78] Tang T. Song J. Li J. Wang H. Zhang Y. Suo H. (2020). A synbiotic consisting of *Lactobacillus plantarum* S58 and hull-less barley β-glucan ameliorates lipid accumulation in mice fed with a high-fat diet by activating AMPK signaling and modulating the gut microbiota. Carbohydr. Polym. 243:116398. doi: 10.1016/j.carbpol.2020.116398, PMID: 32532403

[ref79] van den Berg F. F. van Dalen D. Hyoju S. K. van Santvoort H. C. Besselink M. G. Wiersinga W. J.. (2021). Western-type diet influences mortality from necrotising pancreatitis and demonstrates a central role for butyrate. Gut 70, 915–927. doi: 10.1136/gutjnl-2019-320430, PMID: 32873697 PMC7917160

[ref80] Verma A. Bhagchandani T. Rai A. Nikita Sardarni U. K. Bhavesh N. S.. (2024). Short-chain fatty acid (SCFA) as a connecting link between microbiota and gut-lung axis-a potential therapeutic intervention to improve lung health. ACS Omega 9, 14648–14671. doi: 10.1021/acsomega.3c05846 38585101 PMC10993281

[ref81] Vernia P. Fracasso P. L. Casale V. Villotti G. Marcheggiano A. Stigliano V.. (2000). Topical butyrate for acute radiation proctitis: randomised, crossover trial. Lancet 356, 1232–1235. doi: 10.1016/s0140-6736(00)02787-2, PMID: 11072942

[ref82] Vinolo M. A. Rodrigues H. G. Hatanaka E. Hebeda C. B. Farsky S. H. Curi R. (2009). Short-chain fatty acids stimulate the migration of neutrophils to inflammatory sites. Clin. Sci. (Lond.) 117, 331–338. doi: 10.1042/CS20080642, PMID: 19335337

[ref83] Wang H. B. Wang P. Y. Wang X. Wan Y. L. Liu Y. C. (2012). Butyrate enhances intestinal epithelial barrier function via up-regulation of tight junction protein Claudin-1 transcription. Dig. Dis. Sci. 57, 3126–3135. doi: 10.1007/s10620-012-2259-4 22684624

[ref84] Wang Y. Yao W. Li B. Qian S. Wei B. Gong S.. (2020). Nuciferine modulates the gut microbiota and prevents obesity in high-fat diet-fed rats. Exp. Mol. Med. 52, 1959–1975. doi: 10.1038/s12276-020-00534-2 33262480 PMC8080667

[ref85] Willemsen L. E. Koetsier M. A. van Deventer S. J. van Tol E. A. (2003). Short chain fatty acids stimulate epithelial mucin 2 expression through differential effects on prostaglandin E(1) and E(2) production by intestinal myofibroblasts. Gut 52, 1442–1447. doi: 10.1136/gut.52.10.1442 12970137 PMC1773837

[ref86] Xiao S. Jing S. Jiakui S. Lei Z. Ying L. Han L.. (2022). Butyrate ameliorates intestinal epithelial barrier injury via enhancing Foxp3+ regulatory T-cell function in severe acute pancreatitis model. Turk J Gastroenterol 33, 710–719. doi: 10.5152/tjg.2022.21307, PMID: 35943149 PMC9524497

[ref87] Xiong Y. Ji L. Zhao Y. Liu A. Wu D. Qian J. (2022). Sodium butyrate attenuates taurocholate-induced acute pancreatitis by maintaining colonic barrier and regulating gut microorganisms in mice. Front. Physiol. 17:813735. doi: 10.3389/fphys.2022.813735 PMC8969109 35370779

[ref88] Xu D. Chen V. L. Steiner C. A. Berinstein J. A. Eswaran S. Waljee A. K.. (2019). Efficacy of fecal microbiota transplantation in irritable bowel syndrome: a systematic review and meta-analysis. Am. J. Gastroenterol. 114, 1043–1050. doi: 10.14309/ajg.0000000000000198, PMID: 30908299 PMC7257434

[ref89] Yang N. Lan T. Han Y. Zhao H. Wang C. Xu Z.. (2023). Tributyrin alleviates gut microbiota dysbiosis to repair intestinal damage in antibiotic-treated mice. PLoS One 18:e0289364. doi: 10.1371/journal.pone.0289364, PMID: 37523400 PMC10389721

[ref90] Yehia Kamel M. Zekry Attia J. Mahmoud Ahmed S. Hassan Saeed Z. Welson N. N. Yehia Abdelzaher W. (2023). Protective effect of rivastigmine against lung injury in acute pancreatitis model in rats via Hsp 70/IL6/ NF-κB signaling cascade. Int. J. Immunopathol. Pharmacol. 37:3946320231222804. doi: 10.1177/03946320231222804, PMID: 38112159 PMC10734328

[ref91] Yu S. Xiong Y. Fu Y. Chen G. Zhu H. Mo X.. (2021). Shotgun metagenomics reveals significant gut microbiome features in different grades of acute pancreatitis. Microb. Pathog. 154:104849. doi: 10.1016/j.micpath.2021.104849, PMID: 33781869

[ref92] Yu S. Xiong Y. Xu J. Liang X. Fu Y. Liu D.. (2020). Identification of dysfunctional gut microbiota through rectal swab in patients with different severity of acute pancreatitis. Dig. Dis. Sci. 65, 3223–3237. doi: 10.1007/s10620-020-06061-4, PMID: 32076933

[ref93] Yu C. Zhang Y. Yang Q. Lee P. Windsor J. A. Wu D. (2021). An updated systematic review with meta-analysis: efficacy of prebiotic, probiotic, and synbiotic treatment of patients with severe acute pancreatitis. Pancreas 50, 160–166. doi: 10.1097/MPA.0000000000001734, PMID: 33565792

[ref94] Zhang Y. Chen R. Zhang D. Qi S. Liu Y. (2023). Metabolite interactions between host and microbiota during health and disease: which feeds the other? Biomed. Pharmacother. 160:114295. doi: 10.1016/j.biopha.2023.114295, PMID: 36709600

[ref95] Zhang X. M. Zhang Z. Y. Zhang C. H. Wu J. Wang Y. X. Zhang G. X. (2018). Intestinal microbial community differs between acute pancreatitis patients and healthy volunteers. Biomed. Environ. Sci. 31, 81–86. doi: 10.3967/bes2018.010 29409589

[ref96] Zhao H. B. Jia L. Yan Q. Q. Deng Q. Wei B. (2020). Effect of Clostridium butyricum and butyrate on intestinal barrier functions: study of a rat model of severe acute pancreatitis with intra-abdominal hypertension. Front. Physiol. 29:561061. doi: 10.3389/fphys.2020.561061 PMC7658654 33192557

[ref97] Zhu Y. He C. Li X. Cai Y. Hu J. Liao Y.. (2019). Gut microbiota dysbiosis worsens the severity of acute pancreatitis in patients and mice. J. Gastroenterol. 54, 347–358. doi: 10.1007/s00535-018-1529-0, PMID: 30519748

[ref98] Ziętek M. Celewicz Z. Kikut J. Szczuko M. (2021). Implications of SCFAs on the parameters of the lipid and hepatic profile in pregnant women. Nutrients 13:1749. doi: 10.3390/nu13061749, PMID: 34063900 PMC8224042

